# The effectiveness and safety of Chuna manual therapy on scoliosis: A protocol for systematic review and meta-analysis

**DOI:** 10.1097/MD.0000000000024778

**Published:** 2021-03-05

**Authors:** Seo-Hyun Park, Won-Suk Sung, Sun-Haeng Lee, Yoon-Jae Lee, In-Hyuk Ha, Byung-Kwan Seo, Gyu-Tae Chang, Hoe-Cheon Yang, Dong-Ho Keum, Eun-Jung Kim

**Affiliations:** a College of Korean Medicine, Dongguk University Graduate School, Seoul; b Department of Acupuncture & Moxibustion, Dongguk University Bundang Oriental Hospital, Seongnam-si, Gyeonggi-do; c Department of Clinical Korean Medicine, Graduate School, Kyung Hee University; d Jaseng Spine and Joint Research Institute, Jaseng Medical Foundation, Seoul; e Department of Acupuncture & Moxibustion; f Department of Pediatrics of Korean Medicine, Kyung Hee University Hospital at Gangdong, Seoul; g Korean Society of Chuna Manual Medicine, Seoul, Republic of Korea; h Department of Rehabilitation Medicine of Korean Medicine, Dongguk University Bundang Oriental Hospital, Seongnam-si, Gyeonggi-do; i Department of Acupuncture & Moxibustion, Dongguk University, Seoul, Republic of Korea.

**Keywords:** Chuna manual therapy, meta-analysis, scoliosis, systematic review

## Abstract

**Background:**

Scoliosis is a spinal deformity and is diagnosed as Cobb angle being greater than 10°. Because it is accompanied with structural dysfunction, it can cause pain, worsen the patient's general health and quality of life. The prevalence of scoliosis has been increasing and many treatments, including surgical treatment and conservative treatment, such as observation, bracing, physiotherapy, and Chuna manual therapy (CMT), have been suggested. CMT is a manual therapy in Korean medicine that provides effective stimulation to the patient's body structure to treat structural dysfunction. After Korean national health insurance's coverage of CMT in 2019, the application of CMT for scoliosis has increased, and many studies have been reported. There have been attempts to elucidate the effectiveness of CMT on scoliosis; however, its effectiveness still remains unconfirmed. Therefore, the aim of this study is to evaluate the effectiveness and safety of CMT on scoliosis.

**Methods:**

The published randomized controlled trials that evaluated the effectiveness and safety of CMT for scoliosis will be searched for in multiple electronic databases without the limitation of country and language. Data on characteristics of studies, interventions, comparators, outcome measures, results, and information for assessment of study quality will be extracted. The primary outcome will be the Cobb angle and the secondary outcomes will be the scales of pain, function, quality of life and disability, and adverse events. Data synthesis and analysis will be conducted using the Review Manager software for Windows (RevMan ver. 5.3.; Copenhagen; The Nordic Cochrane Center, The Cochrane Collaboration, 2014). Subgroup analysis to identify the differences between different CMT maneuvers will also be performed. For risk of bias assessment, the “risk of bias” tool from Cochrane Collaboration will be used.

**Results:**

This study will present the clinical evidence on the effectiveness and safety of CMT on scoliosis.

**Conclusion:**

This study will propose useful evidence for treatment, further research, and health policies in the future.

## 1 Introduction

Scoliosis is a spinal deformity involving the deformation of the coronal plane and the loss of normal curvature in the sagittal plane. It causes the vertebrae to curve laterally or deflect from the anatomical central axis with the rotation of the vertebral body; This is defined as the Cobb angle being over 10°.^[1,2]^ According to previous reports in Korea, the prevalence of scoliosis in the thoracolumbar or thoracic region varies from 1.1% to 6% depending on the age group.^[3,4]^ Oh et al reported the prevalence of scoliosis in Korean male was 5.3% and Chang et al reported that the lowest prevalence of scoliosis was individuals aged <10 years at 1.1%, whereas the highest prevalence of scoliosis was, in those age 70 to 79 at 6.1%.^[3,4]^

Scoliosis could be classified into idiopathic scoliosis, congenital scoliosis, neuromuscular scoliosis, degenerative scoliosis, and among others.^[2,5]^ The former 3, idiopathic, congenital, and neuromuscular scoliosis, are usually seen in the pediatric population.^[5,6]^ Among those, the idiopathic form is most common and categorized according to the timing of presentation: infantile, juvenile, and adolescent.^[5–7]^ The congenital scoliosis caused by an embryologic problem during the gestation.^[5,6]^ The neuromuscular form is a result of impairment in upper or lower motor neurons and assorted into neuropathic and myopathic form.^[5,6]^ In recent days, the incidence of degenerative scoliosis has increased and is reported more often in adult patients.^[8–10]^ When there is low back pain or radiating pain in the lower extremities due to intervertebral disc herniation, the patient's trunk may tilt, which can lead to scoliosis.^[2,8–10]^

The goal of treatment for scoliosis varies according to the age and reason.^[2]^ During the growth period, the major goal is to prevent further transformation during the growth process, respiratory dysfunction, and pain emanating from the spine.^[1,2,5]^ Conversely, reducing the pain or other problem caused by scoliosis is more important in adulthood.^[1,2,5]^ The treatments are divided into surgical treatment and conservative treatment, which includes observation, bracing, physiotherapy, taping, Chuna manual therapy (CMT), and exercise, among others.^[1,2]^ Because the surgical treatment has associated risks, including postoperative complications, conservative treatment is widely used for patients with a small Cobb angle.^[1,2,5,10]^ However, conservative treatment also has limitations, such as increasing risk of progression, thoracic insufficiency, chest wall deformity, and pulmonary malfunction.^[1,2,5]^

CMT is a manual therapy in Korean medicine, that treats structural or functional problems by providing effective stimulation to the patient's body structure, such as joints, muscles, tendons, ligaments, and fascia.^[2,11,12]^ It induces therapeutic effects by moving, rearranging, and changing the target joint directly. Moreover, it increases the circulation in soft tissues and improves the elongation to solve joint malfunction. CMT is widely used in Korean medicine and is one of the highly satisfactory treatments for both patients and Korean medicine doctors.^[12]^ After the South Korea's national health insurance offered the coverage of CMT in 2019, both the frequency of use and the satisfaction of CMT have increased.^[12]^ In addition, several articles have reported that CMT is an effective choice for scoliosis because it focuses on structural function.^[2,11,12]^

Although there are various standards, CMT be categorized into bonesetting Chuna therapy and fascia Chuna therapy according to the characteristics of the technique.^[2]^ Based on the Korean health insurance criteria,^[11]^ fascia Chuan therapy is classified as simple Chuna therapy, whereas bonesetting Chuna therapy is classified as complex Chuna therapy^[2,13]^ depending on the presence of thrust. Bonesetting Chuna therapy is a technique that directly targets a joint using the lever effect for a specific anatomical contact point.^[2]^ It is characterized by high-velocity, low-amplitude (HVLA) thrust to exceed the physiological range of motion within the anatomical limits^[2]^ and includes joint manipulative techniques. Conversely, fascia Chuna therapy includes joint mobilization, joint distraction, fascia releasing, and other techniques focusing on soft tissues.^[2]^

As the application of CMT increased, many literature reviews on the effectiveness of CMT on scoliosis were reported, but most of them were narrative reviews and few focused on the size of the effect.^[11,12,14,15]^ Both bonesetting Chuna therapy and fascia Chuna therapy focus on joint insufficiency and dysfunction; however, they are not considered the same because of the different procedures involved. This systematic review and meta-analysis will be conducted to evaluate the effectiveness and safety of CMT on scoliosis. Furthermore, subgroup analyses will be performed to present whether or not there is a difference in effectiveness of bonesetting Chuna therapy and fascia Chuna therapy.

## 2 Methods

### 2.1 Study design

A systematic review and meta-analysis will be conducted in accordance with the preferred reporting items for systemic review and meta-analyses protocols 2015 statement.^[16]^

### 2.2 Ethics

A statement of ethics approval is not required as there will be no requirement of patients or collection of personal information.

### 2.3 Study registration

The protocol was registered in INPLASY (Registration number: INPLASY20211033).

### 2.4 Eligibility criteria

#### 2.4.1 Participants

Studies will be eligible if they include participants who were diagnosed as having scoliosis. Scoliosis is a spinal deformity identified when there is at least a 10° lateral curve of the spine. This study will include all types of scoliosis, such as congenital scoliosis, neuromuscular scoliosis, idiopathic scoliosis, and others.

#### 2.4.2 Types of interventions

The eligible intervention is CMT including chiropractic, osteopathy, Tuina, spinal manipulation, mobilization, myofascial release, and massage, among others. The combined intervention with CMT will be accepted if the other intervention was equally used in both experimental and control groups.

CMT is classified into bonesetting Chuna therapy and fascia Chuna therapy based on the technique procedure. Bonesetting Chuna therapy is characterized by the thrust technique which is the HVLA maneuver directed at the spine or manipulative technique classified Grade V according to Maitland grades of mobilization.^[17]^ Fascia Chuna therapy involves a localized passive force delivered to the joint within normal physiological range of motion. Fascia Chuna therapy includes traction and joint mobilization technique classified Grade I–IV according to Maitland grade.

#### 2.4.3 Types of studies

This study will include the published randomized controlled trials that evaluated the effectiveness of CMT for scoliosis. Case reports, observational studies, and cross-sectional studies will be excluded. Crossover-designed studies will be included, but data of only the first phase will be included.

#### 2.4.4 Outcome measures

The primary outcome will be the Cobb angle. The secondary outcomes will include pain index (e.g. visual analog scale, and numerical rating scale), functional status (e.g. curative rate), quality of life and disability (e.g. questionnaires of the 36-item short form health survey, scoliosis research society-22 outcomes questionnaire, Oswestry disability index, and Roland–Morris disability questionnaire), and adverse events.

#### 2.4.5 Language

There will be no limits on the language.

### 2.5 Information sources and search strategy

The following databases will be searched: MEDLINE, EMBASE, Cochrane Library, China National Knowledge Infrastructure, CiNii, Wanfang database, KoreaMed, Korean medical database, Korean Studies Information Service System, National Digital Science Library, Korea Institute of Science and Technology Information, and Oriental Medicine Advanced Searching Integrated System. The following key terms will be used in combination to develop a search strategy in each electronic database's supported language: (scoliosis OR spinal curve) AND (Chuna manual therapy OR Chuna OR manual therapy OR chiropractic OR osteopathy OR Tuina OR spinal manipulation OR mobilization OR myofascial release OR massage). The literature search strategy is presented in Table 1. The search will be also performed for relevant gray literature sources, reports, and dissertations. Manual searches involving referring to textbooks on CMT and its associated references and contacting authors via their e-mail will also be done, if needed.

**Table 1:** Search strategy for the MEDLINE via PubMed.

No.	Search terms
#1	“scoliosis”[MeSH] OR “scoliosis”[Title/abstract]
#2	“scoliosis”[MeSH] OR “scoliosis”[Title/abstract] OR OR “spinal curve”[Title/abstract] OR “congenital scoliosis”[Title/abstract] OR “musculoskeletal scoliosis”[Title/abstract] “degenerative scoliosis”[Title/abstract] OR “traumatic scoliosis”[Title/abstract] OR “idiopathic scoliosis”[Title/abstract]
#3	“chiropractic”[MeSH] OR “manipulation”[MeSH] OR “Chuna manual therapy”[Title/abstract] OR “Chuna” [Title/abstract] OR “CMT” [Title/abstract] OR “manual therapy” [Title/abstract] OR “chiropractic” [Title/abstract] OR “osteopathy” [Title/abstract] OR “tuina” [Title/abstract] OR “spinal manipulation” [Title/abstract] OR “mobilization” [Title/abstract] OR “myofascial release” [Title/abstract]
#4	#1 AND #2 AND #3

### 2.6 Study selection

**Figure 1:**
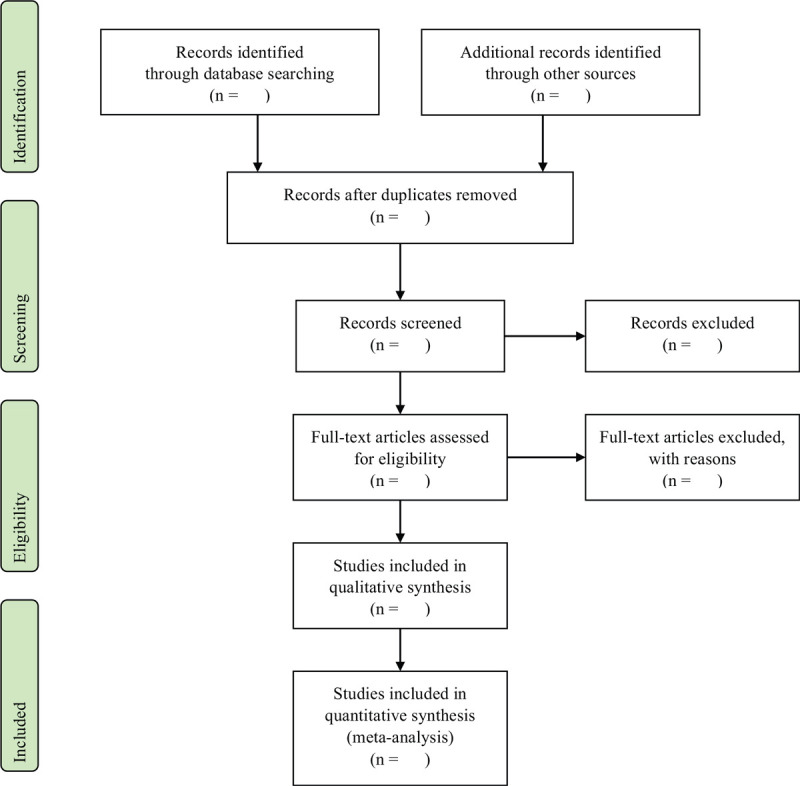
PRISMA flow chart. PRISMA = preferred reporting items for systemic review and meta-analyses.

After conducting the search by intervention, 2 researchers will independently perform the screening procedure. Duplicated studies will be excluded first, and other studies will be excluded based on assessment of title, abstract, and full text. Then, 2 reviewers will obtain and review the full texts of the remaining articles based on the pre-determined inclusion criteria. The characteristic of intervention will be divided into 2 groups; bonesetting Chuna therapy and fascia Chuna therapy. The assortment will be performed by 2 reviewers independently according to the methods of intervention described in the full text. The major criteria for classification will be the existence of thrust technique, which may be presented as the HVLA maneuver directed at the spine, mobilization technique classified Grade V in Maitland grade, or thrust technique. If there is any disagreement in the entire procedure, 2 reviewers will discuss and reach a consensus. If they fail to do so, the final decision will be taken by the third reviewer (Fig. 1).

### 2.7 Data management

The results of the search will be compiled using the management software EndNote X9.

### 2.8 Data extraction

Two reviewers will extract the data according to the predefined criteria. Study characteristics, patient characteristics, interventions and comparators, outcome measures, results, and information for assessment of study quality will be extracted. If it is necessary, the original author will be contacted by e-mail to obtain any missing data. If there are any disagreements or discrepancies, 2 reviewers will solve the problem through discussion. If the disagreement persists, a third reviewer will make final decision.

### 2.9 Data synthesis and analysis

The Review Manager software for Windows (RevMan ver.5.3; Copenhagen; The Nordic Cochrane Center, The Cochrane Collaboration, 2014) will be used for conducting meta-analysis and evaluating risk ratio or standard mean difference. A random-effect model or fixed-effect model with 95% confidence will be selected according to the heterogeneity to calculate the pooled estimates of the effect size. The heterogeneity will be evaluated using Chi-squared and *I*-squared tests: 0% to 40% indicate unimportant heterogeneity, 30% to 60% mean moderate heterogeneity, 50% to 90% present substantial heterogeneity, and 60% to 100% indicate considerable heterogeneity.

After data synthesis and analysis for identifying the effectiveness of CMT on scoliosis, subgroup analysis will be conducted to evaluate whether or not there are differences between bonesetting Chuna therapy and fascia Chuna therapy. Subgroup analyses according to differences in population or intervention characteristics will be also performed, if it is available. Sensitivity analysis will also be conducted, if needed. If the quantitative synthesis is no available, a narrative review will be conducted using available data.

When there are more than 10 identified studies in the meta-analysis, funnel plots will be used for assessing publication bias. The grading of recommendations assessment, development, and evaluation method will be used for rating the quality of evidence for each outcome.^[18]^

### 2.10 Risk of bias assessment

The risk of bias assessment will be done using the “risk of bias” tool from Cochrane Collaboration and will be performed by 2 reviewers independently. The tool has 7 domains: sequence generation, allocation concealment, blinding of participants and personnel, blinding of outcome assessors, incomplete outcome data, and selective outcome reporting and other bias. The risk of bias for each domain will be rated as “low risk,” “high risk,” or “unclear risk.”^[19]^

## 3 Discussion

Scoliosis is accompanied with spinal deformity, and it can lead to structural dysfunction and pain. This problem can affect the patient's general health and quality of life, thus making its management important. Although its prevalence has been increasing and many treatments have been suggested, there are limitations in the evaluation of their effectiveness and safety, especially for CMT. In Korea, many studies have attempted to find out the effectiveness of CMT on scoliosis and other countries also have reported its effects. This study will include all such studies published in Korea, China, and other countries without any restrictions. This study will focus on the effectiveness and safety of CMT on scoliosis. Furthermore, subgroup analysis will be performed to find out whether or not there are differences between bonesetting Chuna therapy and fascia Chuna therapy. To our knowledge, this will be the first attempt at identifying such difference. We hope to present useful evidence for treatment, further research, and health policy in the future.

## Author contributions

**Conceptualization:** Seo-Hyun Park, Dong-Ho Keum.

**Funding acquisition:** Eun Jung Kim.

**Investigation:** Seo-Hyun Park, Won-Suk Sung, Sun-Haeng Lee.

**Methodology:** Seo-Hyun Park, Won-Suk Sung, Yoon-Jae Lee, In-Hyuk Ha.

**Project administration:** Byung-Kwan Seo, Gyu-Tae Chang, Hoe-Cheon Yang, Dong-Ho Keum, Eun Jung Kim.

**Supervision:** Eun Jung Kim.

**Writing – original draft:** Seo-Hyun Park.

**Writing – review & editing:** Seo-Hyun Park, Won-Suk Sung, Eun Jung Kim.

## References

[1] Negrini S Donzelli S Aulisa AG. 2016 SOSORT guidelines: orthopaedic and rehabilitation treatment of idiopathic scoliosis during growth. Scoliosis Spinal Disord 2018;13:3 doi: 10.1186/s13013-017-0145-8. 29435499 10.1186/s13013-017-0145-8 PMC5795289

[2] The Society of Korean Medicine Rehabilitation. Korean Rehabilitation Medicine. 5th ed. Seoul: Kunja; 2020.

[3] Chang DG Kim GU Suk SI. Prevalence of thoracic scoliosisin Koreans using simple chest radiography. J Korean Soc Spine Surg 2019;26:56–62.

[4] Blevins K Battenberg A Beck A. Management of scoliosis. Adv Pediatr 2018;65:249–66. 30053928 10.1016/j.yapd.2018.04.013

[5] Diebo BG Shah NV Boachie-Adjei O. Adult spinal deformity. Lancet 2019;294:160–72. 10.1016/S0140-6736(19)31125-0 31305254

[6] Kim HN Kim HS Moon ES. Scoliosis imaging: what radiologists should know. Radiographics 2010;30:1823–42. 21057122 10.1148/rg.307105061

[7] Kang MS Suh SW Choi SJ. Juvenile idiopathic scoliosis. J Korean Orthop Assoc 2016;51:117–24.

[8] Cho KJ Kim YT Seo BH. Radiological evaluation and classification of adult spinal deformity. J Korean Orthop Assoc 2016;41:1–8.

[9] Cho KJ Kim YT Shin SH. Surgical treatment of adult degenerative scoliosis. Asian Spine J 2014;8:971–81. 10.4184/asj.2014.8.3.371 PMC4068860 24967054

[10] Jung GH Lee H Kong HJ. A systematic review of Chuna manual therapy for adolescent idiopathic scoliosis. J Acupunct Res 2019;36:119–30.

[11] Heo I. Chuna manual therapy for spinal scoliosis: a review of clinical study. J Korean CHUNA Manual Med Spine Nerves 2019;14:39–47.

[12] Hwang DK Im BM Shin YS. A study on evaluation of pilot project for conversion of chuna manual therapy health insurance benefit. [Internet]. Wonju: Health Insurance Review & Assessment Service; 2018. Available at: http://www.alio.go.kr/informationResearchView.do?seq=2410031 [access July 2, 2018].

[13] Lotan S Kalichman. Manual therapy treatment for adolescent idiopathic scoliosis. J Bodyw Mov Ther 2019;23:189–93. 30691751 10.1016/j.jbmt.2018.01.005

[14] Lee SW Chu HY Kim H. A review of the domestic trends of Korean traditional medicine for idiopathic scoliosis. J Korean Med Rehabil 2020;30:55–64.

[15] Taylor P Pezzullo L Grant SJ. Cost-effectiveness of acupuncture for chronic nonspecific low back pain. Pain Pract 2014;14:599–606. 24138020 10.1111/papr.12116

[16] Moher D Shamseer L Clarke M. Preferred reporting items for systematic review and meta-analysis protocols (PRISMA-P) 2015 statement. Syst Rev 2015;4:1 doi: 10.1186/2046-4053-4-1. 25554246 10.1186/2046-4053-4-1 PMC4320440

[17] Hengeveld E, Banks K. (ed) Maitland's Peripheral Manipulation. 4th ed. Elsevier: London; 2005.

[18] Guyatt GH Oxman AD Vist GE. GRADE: an emerging consensus on rating quality of evidence and strength of recommendations. BMJ 2008;336:924–6. 18436948 10.1136/bmj.39489.470347.AD PMC2335261

[19] Higgins JP Altman DG Gøtzsche PC. The Cochrane Collaboration's tool for assessing risk of bias in randomised trials. BMJ 2011;343:d5928. 22008217 10.1136/bmj.d5928 PMC3196245

